# Dr. Sally A. Harris

**Published:** 1913-11

**Authors:** 


Dr. Sally A. Harris, N. Y. Medical College and Hospital for Women, 1878, died at Scarsdale, N. Y., September 29, aged 87. She was formerly a practitioner of White Plains.

